# Exploring Function Prediction in Protein Interaction Networks via Clustering Methods

**DOI:** 10.1371/journal.pone.0099755

**Published:** 2014-06-27

**Authors:** Kire Trivodaliev, Aleksandra Bogojeska, Ljupco Kocarev

**Affiliations:** 1 Department of Intelligent Systems, Faculty of Computer Science and Engineering, Skopje, Macedonia; 2 Macedonian Academy of Sciences and Arts, Skopje, Macedonia; Technische Universität Dresden, Medical Faculty, Germany

## Abstract

Complex networks have recently become the focus of research in many fields. Their structure reveals crucial information for the nodes, how they connect and share information. In our work we analyze protein interaction networks as complex networks for their functional modular structure and later use that information in the functional annotation of proteins within the network. We propose several graph representations for the protein interaction network, each having different level of complexity and inclusion of the annotation information within the graph. We aim to explore what the benefits and the drawbacks of these proposed graphs are, when they are used in the function prediction process via clustering methods. For making this cluster based prediction, we adopt well established approaches for cluster detection in complex networks using most recent representative algorithms that have been proven as efficient in the task at hand. The experiments are performed using a purified and reliable Saccharomyces cerevisiae protein interaction network, which is then used to generate the different graph representations. Each of the graph representations is later analysed in combination with each of the clustering algorithms, which have been possibly modified and implemented to fit the specific graph. We evaluate results in regards of biological validity and function prediction performance. Our results indicate that the novel ways of presenting the complex graph improve the prediction process, although the computational complexity should be taken into account when deciding on a particular approach.

## Introduction

A protein within a cell is rarely a single constituent of the mechanism that performs its function. It has been observed that proteins involved in the same cellular processes often interact with each other [1] making the protein-protein interactions (PPI) fundamental to almost all biological processes [2]. Significant amount of data is produced with the advancement of high-throughput technologies. Yeast-two-hybrid, mass spectrometry, and protein chip technologies have allowed the construction of large interaction networks [3], and are now scaled up to produce extensive genome-wide data sets that are providing us with a first glimpse of global interaction networks. However, these rapid improvements come at the price of a vast majority of known proteins not being experimentally characterized, and their function is yet unknown [4]. As has been commonly realized, the acquisition of data is but a preliminary step, and a true challenge lies in developing effective means to analyze such data and endow them with physical and/or functional meaning [5]. This has prompted the computational function prediction as one of the most challenging problems of the postgenomic era.

PPI data has the nature of networks. This provides a global view of the context of each protein. There is more information in a protein interaction network (PIN) compared to sequence or structure alone. A protein in a PIN is annotated with one or more functional terms. Multiple and sometimes unrelated annotations can occur due to multiple active binding sites or possibly multiple stable tertiary conformations of a protein. The annotation terms are commonly based on an ontology. A major effort in this direction is the Gene Ontology (GO) project [6]. GO characterizes proteins in three major aspects: molecular function, biological process and cellular localization.

We can now characterize the computational function prediction as the process of understanding the relationship between the protein's interaction context and its functions. Grouping proteins of the PIN into sets (clusters) which show greater similarity among proteins in the same cluster than in different clusters has been shown as an effective approach to accomplish this goal [7]. Since biological functions can be carried out by particular groups of proteins, dividing networks into naturally grouped parts (clusters) is an essential way to investigate some relationships between the function and topology of networks or to reveal hidden knowledge behind them. Typical graph clustering methods often result in a poor clustering arrangement [8] so PINs have been weighted based on topological properties such as shortest path length [9], [10] and clustering coefficients [11] in order to achieve an improvement in the clustering results. In [12]– the edge-betweenness and its modified version, using weights generated from micro array expression profiles, have been used as a method to find functional modules in the PIN. A method that combines the results of multiple, independent clustering arrangements into a single consensus cluster structure is presented in [16].

PINs have also been analyzed by extracting protein complexes, i.e. finding densely connected subgraphs within the network. To infer such complexes many methods have been proposed. The Markov Cluster algorithm (MCL) [17] simulates a flow on the graph by calculating successive powers of the associated adjacency matrix. Restricted Neighborhood Search Clustering (RNSC) [18]), is a cost-based local search algorithm that explores the solution space to minimize a cost function, calculated according to the numbers of intra-cluster and inter-cluster edges. Super Paramagnetic Clustering (SPC) [19] is a hierarchical clustering algorithm inspired from an analogy with the physical properties of a ferromagnetic model subject to fluctuation at nonzero temperature. Molecular Complex Detection (MCODE) [20] is based on node weighting by local neighborhood density and outward traversal from a locally dense seed protein to isolate densely connected regions. Detection of highly connected subgraphs (cliques) combined with Monte Carlo optimization is considered in [21]. The authors distinguish two types of clusters: protein complexes and dynamic functional modules. Highly connected subgraphs algorithm is used in [22] for discovery of protein complexes, while the authors of [23] use spectral clustering for generating modules, and possible functional relationships among the members of the cluster for predicting new protein-protein connections. More recent approaches exploit semantic similarity measures based on GO between pairs of proteins within the PIN. PROCOMOSS [24] uses a multi-objective evolutionary approach in which graphical properties as well as biological properties based on GO semantic similarity measure are considered as objective functions for detecting protein complexes in a PIN. CSO [25] performs clustering based on network structure and ontology attribute similarity on GO attributed PINs. Both of these algorithms achieve state-of-the-art performance. These results are another proof that topological features of the PIN alone are insufficient for proper partitioning of the PIN and the network needs to be augmented.

In this paper we address the problem of function prediction in twofold manner. First, we propose novel graph representations of the PIN each having different level of complexity and different inclusion of the annotation information within the graph. Second, we select state-of-the-art algorithms for cluster detection that have not yet been used on PINs and we examine their efficiency in detecting clusters within the different graph representations of the PIN as previously defined. Since we are interested in function prediction the exploration of these methods goes one step further in establishing efficient clustering in terms of accurate cluster based function prediction and establishing the benefits and the drawbacks of combining the methods with the different graph representations of the PIN in the functional annotation process. We conclude the paper with a discussion of what would be the recommended approach of predicting a function in the PIN depending on the priorities of the outcome i.e. what is the best experimental setup if the prediction is done network wide versus a prediction for a single (or a small group of) protein(s), and if the prediction accuracy is of higher importance than its coverage, and vice versa.

## Materials and Methods

### Protein-Protein Interaction Data

High-throughput techniques are prone to detecting many false positive interactions, leading to a lot of noise and non-existing interactions in the databases. Furthermore, some of the databases are supplemented with interactions computationally derived with a method for protein interaction prediction, adding additional noise to the databases. Therefore, none of the available databases are perfectly reliable and the choice of a suitable database should be made very carefully.

**Figure 1:**
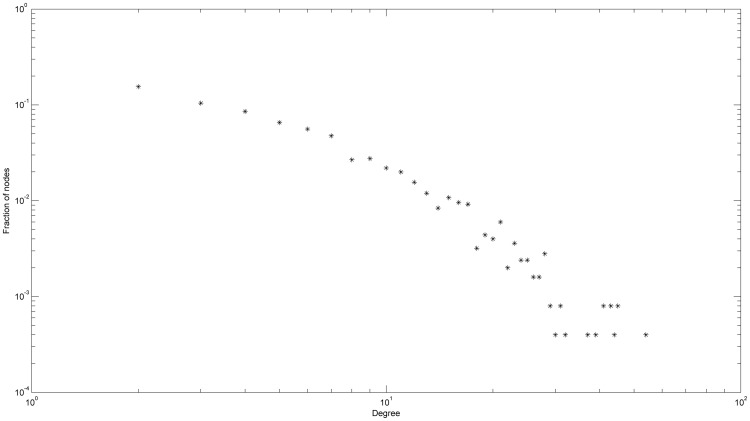
Degree distribution of the primary PIN on log-log scale.

We conduct our experiments on Saccharomyces cerevisiae PPI data which are compiled from a number of established datasets used in previous research on PPI. Namely, we first merge the PPI datasets of Uetz [26], Ito [27], Ho [28], Krogan [29], and Gavin [30]. We then filter out interaction from the merged dataset based on the number of supporting evidence found in DIP [31], MIPS [32], MINT [33], BIND [34] and BioGRID [35]. The resulting dataset contains only protein-protein interactions which have more than one experimental evidence. The functional terms for each protein are taken from the SGD database [36], and are unified with the GO terminology. This data is further purified as proposed in [37]. First, the trivial functional terms, like ‘unknown molecular function’ are erased. Then, additional terms are calculated for each protein by the policy of transitive closure derived from the GO. The extremely frequent terms (appearing as annotations to more than 300 proteins) are also excluded, because they are very general and do not carry significant information. The final dataset is highly reliable and consists of 2502 proteins with 6354 interactions between them and has a total of 888 functional terms and 31515 protein-term pairs. The average node degree of the resulting protein interaction network is 5.08 and the clustering coefficient is 0.18. Figure 1 shows the degree distribution of the network on log-log scale.

### Protein Interaction Network Representation

As previously stated, PPI data has the properties of a network and therefore can be represented as a graph. We introduce several different graph representations of the PIN, each of which represents the information within the data at a different level. Our first goal is to explore the level of detail that is sufficient for effective clustering of the PIN and function prediction, and to show that the novel augmented representations significantly improve performance. We point out here that all graphs resulting from a PIN are undirected since an interaction itself is undirected. The different representations with ascending level of complexity are defined as follows.

#### Simple Graphs

The most basic definition of a PIN graph representation is through *simple graph* with 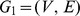 where nodes 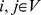 correspond to proteins, and edges 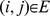 correspond to interaction between “proteins” *i* and *j*. The simple graph is unweighted. With this graph we use only the topology of the PIN to determine clusters. For our data we have 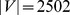 and 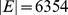.

#### Weighted Graphs

The simplest way to enrich the previous representation is to add weights to edges from *E* and thus define a weighted graph 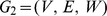 for the PIN, where *W* is a matrix whose elements 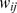 are the weights of the edges 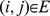. Weights can be calculated in three different ways [38].

- *Content-based weights*: a content-based weight calculation is one that assigns weight 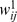 to the edge 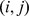 by looking at the terms (“content”) associated with nodes *i* and *j*, not taking their environment (the graph structure) into account. If 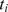 is the set of terms associated with node *i* and 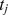 is the set of terms associated with *j*, 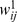 can be computed using the normalized Jaccard Index as follows:
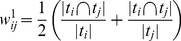
(1)
- *Structure-based weights*: a structure-based weight calculation is one that takes the context of the nodes *i* and *j* into account, but not the content of the nodes themselves, when calculating weight 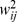 for the edge 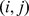. In order to calculate 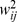 we need to derive a way to map the context of *i* and *j* so that the result contains all the structural information about these nodes. The structural information of the graph 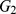 is naturally encoded in its adjacency matrix 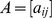 so we can define the weight matrix 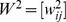 as follows:
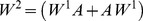
(2)where 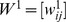 is the content-based weight matrix. Since 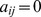, 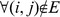, the first part of Eq. 2 gives the sum of all content-based weights of edges between node *i* and all neighbours of *j*, while the second part is the sum of all content-based weights between node *j* and all neighbours of *i*. PINs are known to have proteins that interact with many other, which gives rise to hubs within the graph representing the PIN. Eq. 2 will give high scores to nodes with high degree and vice versa, i.e. low scores to nodes with low degree, so we average the values to overcome this unwanted effect and get Eq. 3. Additionally 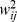 are normalized to be in the same range as 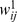.
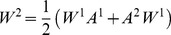
(3)where 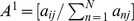, 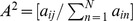, and 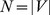.
- *Hybrid weights*: it combines both content-based and structure-based weights; a natural way of combining them is taking the average of the two:
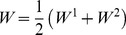
(4)We note that many other ways of defining *W* ^1^ and *W* ^2^ are possible. We are pointing out that multiple definitions of weighting may make sense, and that, depending on the task, one may be more suitable than the other. We will show how the different weighting schemes influence the result of clustering and function prediction.

#### Protein-Term Graphs

**Figure 2:**
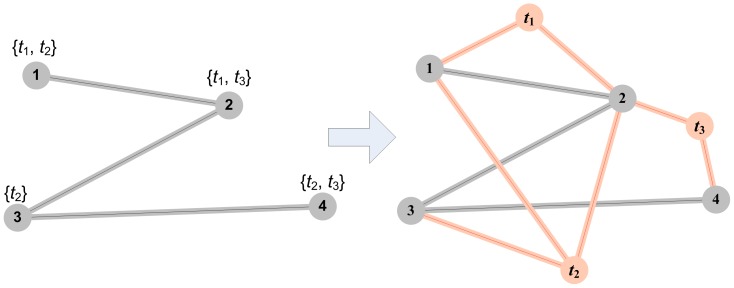
Protein-term graph. Terms associated to proteins and their connections are added to graph. Gray nodes 1, 2, 3 and 4 are proteins and red nodes *t*1, *t*2 and *t*3 are terms. If one node is annotated with one or more terms links to these nodes are added (red links).

We define 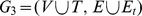 as a *protein-term graph* in which the terms associated to proteins in the PIN become part of its representation. More specifically *T* is the set of all terms present within the PIN and each term *t_i_* is represented as a node in the graph. *E_t_* is the set of edges 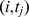 where 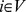, 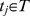 and term *t_j_* is associated with protein *i* in the PIN. This definition of the representation and the set of additional edges *E_t_* takes into account additional edges only between protein nodes (*V*) and new term nodes (*T*), and no edges exist between two term nodes, as shown on Figure 2. *V* and *E* have the same definition as in the previous representations. The graph is unweighted.

In this way functional relationships between the proteins in the PIN are directly included in the graph representation and therefore in the process of clustering and function prediction. When we create the protein-term graph for our data we have a total of 3390 nodes (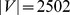, 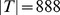) and 37869 edges (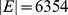, 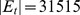).

#### Full Functional Connected Graphs

The *full functional connected* (FFC) graphs are defined as 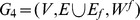. Let *t_i_* and *t_j_* be the sets of terms associated with nodes *i* and *j*, respectively, then for edge 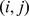 we have 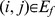 if and only if 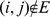 and 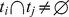. 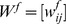 is the weighted matrix. In other words if two proteins in the PIN share a term, an edge is added in the graph between them even if they don't interact together, thus creating “false” interactions. However the information for the “true” interactions is preserved through the weight matrix. Namely, each edge is assigned a content-based weight, with an additional constant being added to edges representing real interactions. Formally we have:
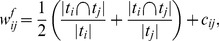
(5)where
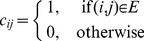
(6)for every 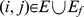. We take the constant to be 1 since that is the maximum value of the content-based weight in the case of identical terms in the two connected nodes. This way we ensure that each true interaction weight is larger (or equal in the worst case) than any false interaction weight, but in the same time allowing the content similarity to have at most the same effect as a true interaction. The FFC graph for our PIN has a total of 1086948 edges (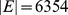, 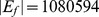).

### Clustering Algorithms

The modern science of networks has brought significant advances to our understanding of complex systems, with the organization of the vertices in clusters (also referred to as communities) being one of the most relevant features of the graphs representing such systems. The problem of detecting clusters is very hard and not yet satisfactory solved, and is in the focus of a large interdisciplinary scientific community [39]. PINs are complex networks, and as such communities (corresponding to functional modules and complexes) emerge in their graph representations [10]. In our work we focus on most recently developed methods for cluster detection in graphs which have been classified as most efficient [40]. These algorithms are initially employed in detecting community structure in different real-life networks and to our knowledge have not yet been used in clustering PINs. Taking this into account our motivation and goal is to explore how these state-of-the-art algorithms perform when used in a PIN, and even further explore how the combination with the different PIN representations affect the function prediction performance.

#### Modularity Function Algorithms

One of the biggest breakthroughs in cluster detection was the Girvan and Newman modularity function [41]. They propose an equation that calculates the quality of a given clustering compared to a corresponding random graph. The randomization of the edges is done with preserving each node degree. The modularity function is defined as:
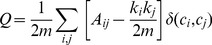
(7)The term *A_ij_* has different meaning for different graph representations. When we work with unweighted graphs 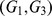 the term is the corresponding member of the adjacency matrix (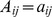), while in weighted graphs 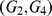 the term is the corresponding member of the weight matrix (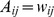) since these graphs are a simple generalization [42]. Terms *k_i_* and *m* are defined with 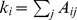 and 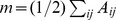, and in the case of unweighted graphs correspond to node degree and total number of nodes, respectively. The probability of an edge existing between nodes *i* and *j* if connections are made at random but respecting node degrees is 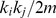, *c_i_* defines the cluster to which node *i* is attached and 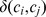 is the Kronecker delta symbol where 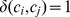 if 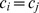 and 0 otherwise. This function gives the difference of the fraction of edges that fall into the cluster and the expected number of edges distributed at random. A value less than 1 means that the number of edges in the group is greater than the number at random i.e. the cluster is well defined, and otherwise, values between zero and −1 mean that the analysed edges do not form good cluster.

The “Fast Community” (FC) [43] community structure inference algorithm is based on a greedy technique that maximizes the Girvan and Newman modularity function. The algorithm uses hierarchical agglomerative method where at the beginning each node represents one cluster. Nodes and later clusters are merged trying to maximize the modularity exploring the full topology of the graph. The novelty of this algorithm is the usage of data structures for sparse matrices, max-heaps, that make this algorithm much faster and suitable for analysis of large graphs.

The proposed algorithm from Blondel et al. (BGLL) [44] uses a different greedy technique using supervertices for representation of the communities and calculating the modularity. At start all nodes are in different clusters but as each node chooses a new cluster the clusters are replaced with supervertices. Two supervertices are connected if there exists an edge between any two nodes from the two supervertices. Again at each step the modularity is calculated from the initial topology. This algorithm finds maximum modularity better than the algorithm used by Clauset et al. [43] but its limitation is in the storage demands.

#### Multi-Resolution Algorithms

Recently it has been shown that modularity optimization may fail to identify clusters smaller than a scale which depends on the total number *N* of links of the network and on the degree of interconnectedness of the clusters, even in cases where clusters are unambiguously defined, characterizing these methods with a so called resolution limit [45]. A new class of methods that deals with this problem is based on multi-scale quality functions. These quality functions incorporate a resolution parameter allowing to tune the characteristic size of the clusters in the optimal partition and aim at uncovering modules at the true scale of organization of a network, i.e., not at a scale imposed by modularity optimization. The publication of Lambiotte [46] gives good overview of the existing multi-resolution quality functions also presenting a new method that tries to unify them by looking into the dynamics of the partitioning problem. The key idea is to measure the quality in terms of stability of module associated to a stationary Markov process modeled as a random walk process. The resulting quality function for detecting modules on multiple-scales is defined as follows:
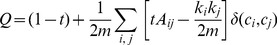
(8)where *t* represents the time parameter of the random walk, equivalent to the Hamiltonian introduced by Reichard and Bornhodt [47]. This equation is the same as the modularity function (7) when the time parameter *t* is equal to 1. The algorithm implementation suggested in [46] and [48] uses the same greedy technique for modularity maximization as in [44]. We performed experiments for the time parameter ranging from 1 to 10 (as suggested in [48]) and we obtained the best results when the parameter equals 5. We'll refer to this algorithm with time parameter set to 5 as TimeBGLL.

#### Edge Clustering Algorithms

Partitioning of nodes in a graph has the disadvantage of being incompatible with the existence of overlapping clusters, i.e. situations where nodes belong to several clusters. This overlap is known to be present at the interface between clusters, but can also be pervasive in the whole graph [49]. In these situations a partition of the nodes is questionable as it imposes undesired constraints on the cluster detection problem. Since edges in the graphs representing the PINs often correspond to one particular type of interaction in the PIN, they typically belong to one single cluster. Therefore we define clusters as partitions of edges rather than of nodes. The edges incident at a single node may belong to several partitions and in this sense, nodes can be members of several clusters.

We adopt the method proposed in [50] since it naturally fits the problem at hand, and also can deal with weighted graphs as described in [51]. Without losing generality we can assume the definition 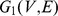 for an unweighted node graph. The method first transforms 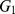 in an unweighted line graph 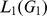 and then uses random walk dynamics to measure the quality function. In principle, any node clustering algorithm can be used. However since optimisation of modularity is related to the behaviour of random walkers on a graph and the construction of 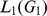 preserves the dynamics of random walkers, it makes sense to apply the modularity optimisation approach to find the partitions of the line graph 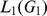. We use the modularity maximization algorithm proposed in [44].

The conversion of the graph from node to line is done as follows: first the node graph is represented using the incidence matrix 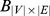, where 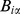 is equal to 1 if edge *α* is related to node *i* and 0 otherwise. The matrix *B* can be seen as an adjacency matrix of a bipartite network. The line graph is constructed with projection of the bipartite graph by taking all nodes of one type for the nodes of the projected graph. A link is added between two nodes in the projected graph if two nodes have at least one node of the other type in common in the original bipartite graph, resulting in the adjacency matrix 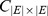 of the line graph 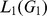, with elements defined by:
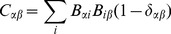
(9)where 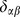 is the Kronecker delta symbol.

By calculating the adjacency matrix as in Eq. 9 nodes with high degree, hubs, are given too much prominence in the line graph, so normalization is used to avoid this effect and 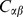 is calculated with:
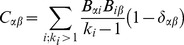
(10)where *k_i_* is the degree of node *i*.

**Figure 3:**
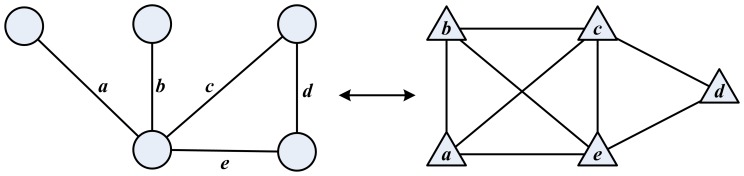
The figure shows the transformation from node graph (on the left) to corresponding line graph (on the right). Edges *a*, *b*, *c*, *d*, and *e* from the node graph are mapped to nodes *a*, *b*, *c*, *d*, and *e* on the line graph, respectively.

When we work with weighted node graphs, 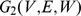, a second weighted incidence matrix 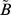 is introduced, where 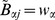 if edge *α* is incident on vertex *j* and has weight 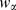. Each node *i* has strength 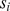, defined as the sum of all weights of its incident edges. As in the unweighted case the normalized adjacency matrix is computed for the weighted line graph 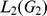 given with:
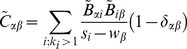
(11)The visual representation of the node to line graph transformation is shown on Figure 3.

#### Random Walks and Maps Algorithms

The ability of random walks to generate dynamics and represent information flow in the network makes them suitable for usage in the clustering problem. Probability flow of random walks on graph are used for creation of efficient and accurate clustering method by Rosvall and Bergstrom (Infomap) [52]. This algorithm additionally uses Huffman coding to describe the path on the network that also allows compression of the maps and speeding up the module detection. Using this coding retention of the unique names of the important structures formed during the random walks is provided. The random walk equation used for undirected graphs is as follows:
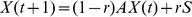
(12)where in the case of unweighted graphs 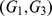, *A* is the normalized adjacency matrix, while in the case of weighted graphs 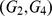, *A* is the weight matrix *W*, *r* is teleportation or restart probability, *X*(*t*) is the probability vector for the random walker visiting a node at time *t*, and *S* is the starting probability vector (usually *S* is all zeros except start node value equal 1). At beginning *X*(0) = *S*.

### Functional Annotation

There are few different methods in the literature for assigning terms to a query protein after clusters are determined. Each of the methods is based on calculating a score for each term associated with a node that belongs to the same cluster as the query node, and assigning to the query protein those terms that have a score greater or lower than a predefined threshold depending on the score type being used. In our work we tested hypergeometric enrichment P-value, chi-square statistics and terms frequency within the cluster as scores for predicting terms.

The hypergeometric enrichment 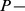 value for term *t* is calculated with:
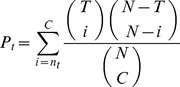
(13)where *N* is the number of nodes in the graph representing the PIN, *T* is the number of nodes in the graph that have term *t* assigned to them, *C* is the cluster size and 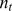 is the number of nodes in the cluster that have term *t* assigned to them. The terms enriched within the cluster (i.e. obtaining 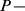 value below some threshold) are then predicted for the query node.

The chi-square statistics score for term *t* is defined with:
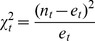
(14)where 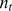 has the same meaning as in the previous score and 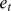 is the expected number of nodes in the cluster that have term *t* assigned to them. The expected number is calculated using simple proportion 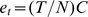, with *T*, *N*, and *C* having the same meaning as in the previous score.

The simplest and most intuitive score calculation approach would be that each term is ranked by its frequency of appearance as a term assigned to nodes within the cluster. This approach is derived from the well known Majority Algorithm used in [53], where a node is assigned with the most frequent terms occurring in its neighbours. Our definition expands the node neighbourhood not only to the direct neighbours but to all nodes that are in the cluster it belongs to, *K*:
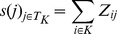
(15)where *T_K_* is the set of terms present in the cluster *K*, and
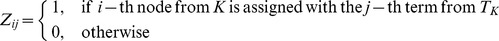
(16)We need to note here that when we work with graph representation *G*_3_, i.e. the protein-term graph, the definition of some quantities used in the score calculations need to be altered. Namely, we say that a term *t* is present in a cluster if the corresponding term node *t* belongs to the cluster. The total number of nodes in the graph corresponds to the total number of protein nodes, the size of the cluster corresponds to the number of protein nodes in the cluster, the number of nodes in the graph with term *t* assigned to them corresponds with the degree of term node *t*, and the number of nodes in a cluster with term *t* assigned to them corresponds to the number of edges between term node *t* and protein nodes belonging to the cluster. For the frequency score *T_K_* is now a set of term nodes and *Z_ij_* is defined with:
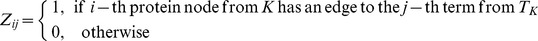
(17)Our experiments showed that the frequency based score for function prediction outperforms the other two scores for any combination of graph representation and clustering algorithm so for simplicity all the results presented are based on this approach.

## Results and Discussion

We tested representative algorithms of the previously described clustering algorithms classes, including FC [43], BGLL [44], TimeBGLL [48], EdgeCluster [50], [51], and Infomap [52]. We performed evaluation of the clustering validity of the different algorithms used. Each of these algorithms was used to determine clusters in each of the different graph representations of our Saccharomyces cerevisiae PIN. We evaluated the clustering results in terms of functional validity and also in terms of accuracy when used in function prediction.

Before we proceed to the results and the discussions for the main focus of this paper, i.e. the function prediction via clustering methods, we give a summary of the computational complexity of our experiments. Although resources are vast nowadays, complexity should not be ignored when deciding upon an experimental setup. Table 1 gives a summary of the sizes of the proposed graph representations of our PIN which is crucial for the expected runtime i.e. computational complexity of the clustering algorithms which is given in Table 2. As can be seen BGLL, TimeBGLL, EdgeCluster and Infomap have essentially linear runtime proportional to the number of edges within the graph, while FC runs in quasilinear time proportional to the number of nodes within the graph, but nevertheless runs faster than any polynomial with exponent strictly greater than 1.

**Table 1:** Summary table for the size of the different proposed graph representations of our PIN.

Graph representation	Number of nodes	Number of edges
Simple	2502	6354
Weighted	2502	6354
Protein-Term	3390	37869
Full Functional Connected	2502	1086948

**Table 2:** Summary for the different clustering algorithms used in this paper showing their computational approach and complexity, where *v* is the number of nodes in the graph being clustered, and *e* is the corresponding number of edges.

Clustering Algorithm	Computational Approach	Complexity
FC	modularity maximization using max heaps	*O*(*v*log^2^ *v*)
BGLL	modularity maximization using multi-passes and supervertices	*O*(*e*)
TimeBGLL	modularity maximization with resolution parameter corresponding to the time parameter of a random walk on the graph	*O*(*e*)
EdgeCluster	modularity maximization on the line graph	*O*(*e*)
Infomap	Minimal description length of a random walker using Huffman coding for each node	*O*(*e*)

### Clustering Validation

Clustering validation was performed using a synthetic benchmark graph as given in [54] in order to compare the different clustering methods used in our work. The synthetic graph was modeled with the parameters of the simple graph representation of our PIN. Since the aim of this experiment is to determine the clustering power of our chosen algorithms and compare them among themselves and with other algorithms used in previous research the graph representation is of no significance and any one can be used. The resulting clusters were compared with the a priori known clusters using the Normalized Mutual Information (NMI) method proposed in [55]. It is based on defining a confusion matrix **M**, where the rows correspond to the “real” clusters, and the columns correspond to the “found” clusters. The element of **M**, 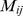 is the number of nodes in the real cluster *i* that appear in the found cluster *j*. A measure of similarity between the clusters, based on information theory, is then:
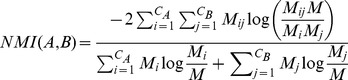
(18)where the number of real clusters is denoted 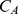 and the number of found clusters is denoted 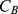, the sum over row *i* of matrix *M* is denoted 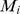, the sum over column *j* is denoted 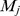 and the total number of nodes is *M*. The normalized mutual information equals 1 if the clusters are identical and 0 if they are totally independent. The definition of the measure when the clusters are overlapping (EdgeCluster) is given in details in the appendix of [56].

Table 3 shows the resulting values for the NMI score calculated as previously explained. These results justify the selected representative clustering algorithms in this paper as they outperform the algorithms, as cited in the introduction, previously used in clustering of PINs based on the topological features of the network, i.e. MCL, RNSC, SPC, and MCODE. Later experiments show that the performance “ranking” on function prediction more or less follows the one given in Table 3.

**Table 3:** NMI values expressing the quality of clustering of a synthetic graph modeled with the parameters of our PIN achieved by employing the clustering algorithms used in this paper (Infomap, TimeBGLL, EdgeCluster, BGLL, FC) and the algorithms previously used in clustering of PINs (MCL, RNSC, MCODE, SPC), as cited in the introduction.

Clustering Algorithm	NMI
Infomap	0.9916
TimeBGLL	0.9062
EdgeCluster	0.8732
BGLL	0.8514
FC	0.8230
MCL	0.4979
RNSC	0.4562
MCODE	0.2360
SPC	0.2147

### Biological Validity of the Clusters

We use many different clustering algorithms that produce different clusters by size and structure for which we evaluate biological relevancy, in other words we test to confirm that the cluster structure has not arisen by chance. If a cluster is biologically relevant, the genes belonging to the same cluster should have similar biological functions [8]. Therefore the functional homogeneity of a cluster is an indicator for its biological validity. Most of the methods for calculating a clusters functional homogeneity include some form of the 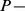 value measure. In [21] a modified 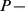 value, which combines computationally derived clusters with “real” complexes derived from the protein databases, is used:
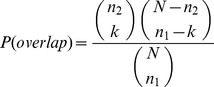
(19)where *N* is the total number of nodes in the network, *n*_1_ and *n*_2_ are the sizes of the two complexes (the derived and the real one), and *k* is the number of nodes they have in common. This measure is effective and good when evaluating a single clustering algorithm but for two or more algorithms the evaluation is time consuming as it requires extraction of the corresponding real complexes for each computed cluster.

A more efficient way of testing functional homogeneity is through functional entropy. The entropy is calculated as the sum of the appearance frequencies of all function terms in the cluster, and multiplies the logarithm of those frequencies [57]:
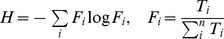
(20)where 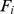 is the appearance frequency of the term *i*, given with the equation above, 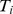 is the number of times that term appears in the clusters and *n* is the number of distinct terms present in the cluster. If the nodes in the same cluster have consistent terms, the value of the functional entropy will be low, being zero when nodes have only one term. We performed the biological validation of our clustering algorithms using entropy. We retained only clusters with more than 2 nodes, and for each combination of graph representation and clustering algorithm we calculated the average entropy over all clusters.

The calculated entropy values are shown in Table 4. Taking into account the definition of the entropy measure lower values would yield an algorithm which is more stringent at identifying functionally coherent clusters. A second and more interesting aspect of the entropy in relation to our research is the correlation of the entropy values and the results of the functional annotation of proteins using the clustering algorithms. Namely, the lower the entropy of an algorithm, the coverage of the average cluster is smaller. The coverage of a cluster here is defined as the ratio between the number of terms present in the cluster and the number of terms present in the whole network. The lower coverage clusters lead to fewer mistakes being made during the term assignment process, but on the downside these clusters may lack the necessary terms needed for correct and complete annotation of a query protein. In terms of the definitions used for the annotation validation this would mean that lower entropy values yield lower False Positives (FPs), but higher False Negatives (FNs). The inverse holds for higher entropy values.

**Table 4:** The entropy values as defined with Eq. 20 for each combination of the PIN graph representation and a clustering algorithm.

Representation Clustering Alg.	Simple	Weighted Content	Weighted Structure	Weighted Hybrid	Protein-Term	FFC
Infomap	0.2528	0.3034	0.3018	0.3002	0.3156	0.5361
TimeBGLL	0.3064	0.3381	0.3271	0.3213	0.5832	0.5783
EdgeCluster	0.2953	0.3294	0.3216	0.3172	0.5716	0.6713
BGLL	0.2707	0.3113	0.3027	0.2993	0.5613	0.6472
FC	0.2807	0.3121	0.3042	0.3001	0.5589	0.6452

Lower values yield smaller coverage of the average cluster, i.e. fewer mistakes during the term assignment process, but on the downside the necessary terms for complete annotation of a query protein may be lacking. In terms of the definitions used for the annotation validation this would mean that lower entropy values yield lower False Positives (FPs), but higher False Negatives (FNs). The inverse holds for higher entropy values.

### Annotation Validation

The effective evaluation of protein functional annotation is challenging. The lack of agreed measures and benchmarks used for assessment of the methods performance makes this task difficult. In our work we used the leave-one-out method when only one protein at time plays the role of a query protein. In the leave-one-out method a random annotation protein is selected and is considered as unannotated. This assumption for no terms present at the query protein affects different representations in different ways. For the unweighted representations no additional changes have to be made, while weighted graphs should be altered since the weight computation is no longer possible as defined by the corresponding equations. Specifically if the representation uses the content based weight its value is substituted with the structure based weight and everything else remains the same. For the Protein-Term representation (*G*_3_) the unannotated query protein assumption means that all edges to term nodes should be deleted. Once the clustering algorithm has been applied, for each term present in the query cluster (i.e. the cluster of the query protein) we calculate its rank according to Eq. (15), and all ranks are then normalized to a range between 0 and 1. We should also note here that when the unannotated query protein assumption causes changes within the graph representation the clustering algorithm should be run for each query protein. The query protein is annotated with all functions that have rank above a previously determined threshold *ω*. For example, for *ω* = 0, the query protein is assigned with all the functions present in its cluster. We change the threshold in the [0,1] range and compute the numbers for the four possible different classes which can occur during the assignment process:

- True Positive (TP): When annotation is assigned and is part of the true annotation set
- True Negative (TN): When annotation is not assigned to the protein and is not part of the true annotation set
- False Positive (FP): When annotation is assigned but is not part of the true annotation set
- False Negative (FN): When annotation is not assigned but is part of the true annotation set

Each annotation is assigned to one of the four classes. Using the number of annotations in each class (given in brackets above) we can calculate the following statistical measures:
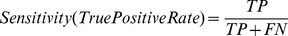
(21)

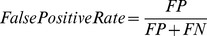
(22)Graphed as coordinate pairs, the Sensitivity and the FalsePositiveRate form the Receiver Operating Characteristic curve (or ROC curve). The ROC curve describes the performance of a model across the entire range of classification thresholds. The Area Under Curve (AUC) of a classifier is equivalent to the probability that the classifier will rank a randomly chosen positive instance higher than a randomly chosen negative instance [58].

**Figure 4:**
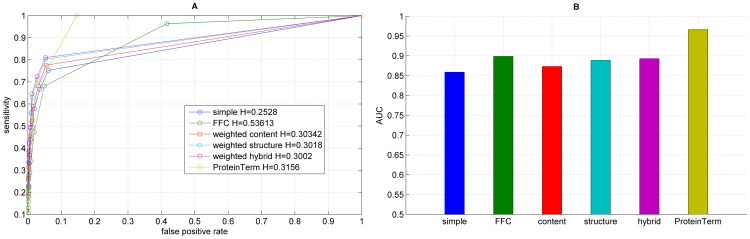
Results for the functional annotation for each graph representation using Infomap, showing the ROC curve (A) with H indicating the corresponding entropy value and the corresponding AUC values (B).

**Figure 5:**
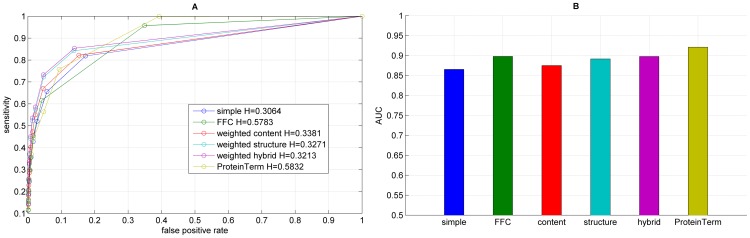
Results for the functional annotation for each graph representation using timeBGLL, showing the ROC curve (A) with H indicating the corresponding entropy value and the corresponding AUC values (B).

**Figure 6:**
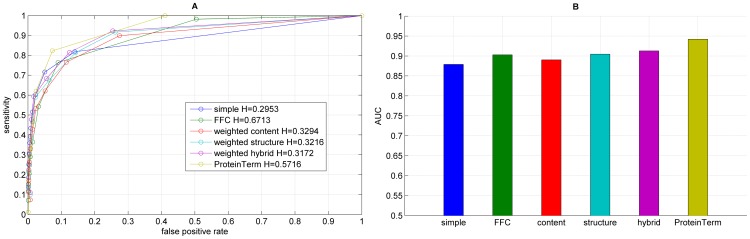
Results for the functional annotation for each graph representation using edgeCluster, showing the ROC curve (A) with H indicating the corresponding entropy value and the corresponding AUC values (B).

**Figure 7:**
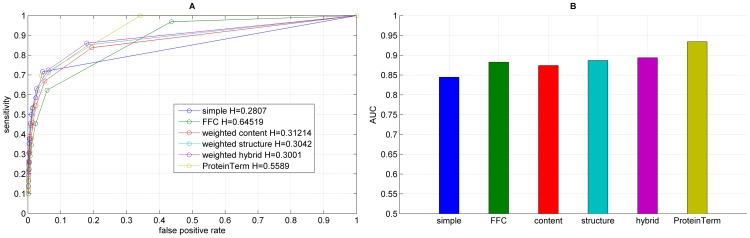
Results for the functional annotation for each graph representation using BGLL, showing the ROC curve (A) with H indicating the corresponding entropy value and the corresponding AUC values (B).

**Figure 8:**
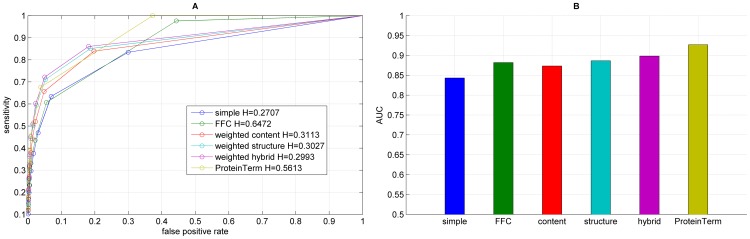
Results for the functional annotation for each graph representation using FC, showing the ROC curve (A) with H indicating the corresponding entropy value and the corresponding AUC values (B).

We performed functional annotation for each combination of a clustering algorithm and a graph representations of our Saccharomyces cerevisiae PIN. Figures 4–8 show the ROC curves and the AUC values for each graph representation for Infomap, timeBGLL, edgeCluster, BGLL and FC, respectively. Tables 5–9, show the sensitivity and false positive rate at threshold values from *ω* = 0 to *ω* = 0.9 with 0.1 step.

**Table 5:** Values for the sensitivity (sens.) and the false positive rate (fpr), for the functional annotation for each graph representation using Infomap, at different threshold values (*ω*).

Threshold/Representation		*ω* = 0	*ω* = 0.1	*ω* = 0.2	*ω* = 0.3	*ω* = 0.4	*ω* = 0.5	*ω* = 0.6	*ω* = 0.7	*ω* = 0.8	*ω* = 0.9
Simple	sens.	0,7523	0,6676	0,5789	0,4952	0,4397	0,3691	0,3307	0,2796	0,2306	0,181
	fpr	0,0614	0,0358	0,0191	0,0133	0,0083	0,0048	0,0039	0,0028	0,0019	0,0014
Weighted Content	sens.	0,7757	0,6818	0,5901	0,5203	0,4549	0,3913	0,3604	0,3133	0,2562	0,1996
	fpr	0,0547	0,0309	0,0163	0,0115	0,0071	0,0042	0,0034	0,0024	0,0017	0,0013
Weighted Structure	sens.	0,8034	0,7123	0,6301	0,5563	0,491	0,4204	0,3805	0,3342	0,2633	0,2136
	fpr	0,0536	0,0283	0,0133	0,0109	0,0069	0,0038	0,0031	0,0023	0,0012	0,0011
Weighted Hybrid	sens.	0,8104	0,7254	0,6457	0,561	0,4951	0,4234	0,3831	0,3363	0,2651	0,224
	fpr	0,0531	0,0277	0,0124	0,0098	0,0064	0,0038	0,0031	0,0021	0,0012	0,001
Protein-Term	sens.	0,9999	0,6804	0,5249	0,4375	0,378	0,3076	0,2733	0,2154	0,1709	0,1295
	fpr	0,1456	0,0303	0,0143	0,0094	0,0066	0,0045	0,0035	0,0022	0,0016	0,0011
FFC	sens.	0,9636	0,6813	0,4731	0,3411	0,2884	0,223	0,1922	0,158	0,1319	0,1071
	fpr	0,4164	0,0493	0,0187	0,0094	0,0059	0,0035	0,0025	0,0016	0,0012	0,001

**Table 6:** Values for the sensitivity (sens.) and the false positive rate (fpr), for the functional annotation for each graph representation using timeBGLL, at different threshold values (*ω*).

Threshold/Representation		*ω* = 0	*ω* = 0.1	*ω* = 0.2	*ω* = 0.3	*ω* = 0.4	*ω* = 0.5	*ω* = 0.6	*ω* = 0.7	*ω* = 0.8	*ω* = 0.9
Simple	sens.	0,8185	0,6562	0,5205	0,428	0,3565	0,2972	0,2453	0,1904	0,1446	0,1153
	fpr	0,1724	0,057	0,0275	0,0156	0,0094	0,0061	0,0041	0,0026	0,0016	0,0011
Weighted Content	sens.	0,8220	0,6701	0,5499	0,4723	0,4027	0,3394	0,2852	0,2288	0,1836	0,1401
	fpr	0,1525	0,0456	0,0221	0,014	0,0087	0,0056	0,0042	0,0028	0,0018	0,0012
Weighted Structure	sens.	0,8426	0,7231	0,5761	0,5234	0,4421	0,3671	0,3247	0,2529	0,2003	0,1594
	fpr	0,1376	0,0473	0,023	0,0132	0,0079	0,0048	0,0039	0,0023	0,0014	0,0011
Weighted Hybrid	sens.	0,8539	0,7328	0,5857	0,5357	0,4506	0,3733	0,3292	0,2571	0,2044	0,1634
	fpr	0,1389	0,0464	0,0225	0,0133	0,0084	0,0051	0,0042	0,0024	0,0015	0,0011
Protein-Term	sens.	0,9995	0,7582	0,5635	0,4433	0,369	0,2971	0,2544	0,2012	0,1639	0,118
	fpr	0,3927	0,0934	0,0474	0,0171	0,0112	0,0071	0,0054	0,0033	0,0018	0,0011
FFC	sens.	0,9573	0,6165	0,4544	0,3535	0,3001	0,2494	0,2084	0,1836	0,1546	0,1145
	fpr	0,3492	0,0416	0,016	0,0078	0,0049	0,0031	0,0021	0,0014	0,001	0,0007

**Table 7:** Values for the sensitivity (sens.) and the false positive rate (fpr), for the functional annotation for each graph representation using edgeCluster, at different threshold values (*ω*).

Threshold/Representation		*ω* = 0	*ω* = 0.1	*ω* = 0.2	*ω* = 0.3	*ω* = 0.4	*ω* = 0.5	*ω* = 0.6	*ω* = 0.7	*ω* = 0.8	*ω* = 0.9
Simple	sens.	0,8184	0,7163	0,6021	0,5154	0,434	0,3604	0,3028	0,2513	0,1854	0,1374
	fpr	0,1403	0,0503	0,0228	0,0137	0,0085	0,0053	0,0036	0,0025	0,0016	0,0011
Weighted Content	sens.	0,8992	0,7654	0,6212	0,5328	0,4282	0,3312	0,2636	0,1713	0,1134	0,0737
	fpr	0,274	0,1147	0,0513	0,0238	0,0139	0,0077	0,0043	0,0025	0,0012	0,0007
Weighted Structure	sens.	0,9171	0,8142	0,6762	0,5888	0,4674	0,3845	0,3018	0,2153	0,1437	0,0997
	fpr	0,2613	0,1429	0,0634	0,0219	0,0142	0,007	0,004	0,0022	0,0011	0,0007
Weighted Hybrid	sens.	0,9232	0,8152	0,6834	0,5933	0,4779	0,3931	0,3077	0,2246	0,1525	0,1099
	fpr	0,2537	0,1242	0,0553	0,0172	0,0102	0,0069	0,0038	0,0021	0,001	0,0007
Protein-Term	sens.	0,9997	0,8233	0,6203	0,4623	0,3272	0,2918	0,2191	0,1744	0,1491	0,0118
	fpr	0,4104	0,0732	0,0242	0,0128	0,0072	0,0056	0,0045	0,0028	0,0014	0,0009
FFC	sens.	0,9823	0,7644	0,5430	0,3648	0,29	0,2483	0,2045	0,1521	0,1194	0,0710
	fpr	0,5042	0,0902	0,0319	0,0138	0,0071	0,0049	0,0037	0,0018	0,001	0,0009

**Table 8:** Values for the sensitivity (sens.) and the false positive rate (fpr), for the functional annotation for each graph representation using BGLL, at different threshold values (*ω*).

Threshold/Representation		*ω* = 0	*ω* = 0.1	*ω* = 0.2	*ω* = 0.3	*ω* = 0.4	*ω* = 0.5	*ω* = 0.6	*ω* = 0.7	*ω* = 0.8	*ω* = 0.9
Simple	sens.	0,7166	0,5843	0,5338	0,4999	0,4542	0,3776	0,3523	0,3049	0,2619	0,2199
	fpr	0,0447	0,0238	0,0142	0,0106	0,0075	0,0041	0,0034	0,0027	0,0021	0,0019
Weighted Content	sens.	0,8381	0,6699	0,5455	0,4579	0,3798	0,3112	0,2627	0,2096	0,169	0,116
	fpr	0,1942	0,0515	0,0246	0,0154	0,0098	0,0061	0,0044	0,0028	0,002	0,0013
Weighted Structure	sens.	0,8524	0,7123	0,6209	0,5235	0,4323	0,3653	0,3003	0,2399	0,1872	0,1348
	fpr	0,1833	0,0632	0,0287	0,0143	0,0095	0,0058	0,004	0,0023	0,0015	0,001
Weighted Hybrid	sens.	0,8615	0,7256	0,6299	0,5319	0,4432	0,3749	0,3087	0,2483	0,1955	0,141
	fpr	0,1786	0,0614	0,0253	0,0139	0,0095	0,0058	0,0039	0,0023	0,0015	0,001
Protein-Term	sens.	0,9992	0,6978	0,5343	0,4478	0,3837	0,3292	0,3079	0,232	0,1784	0,1204
	fpr	0,3416	0,041	0,0187	0,0104	0,0073	0,0057	0,0046	0,003	0,0021	0,0011
FFC	sens.	0,9693	0,6233	0,4538	0,3466	0,2907	0,2567	0,2234	0,1606	0,135	0,0989
	fpr	0,4361	0,0582	0,0236	0,011	0,0066	0,0049	0,0038	0,002	0,0018	0,0011

**Table 9:** Values for the sensitivity (sens.) and the false positive rate (fpr), for the functional annotation for each graph representation using FC, at different threshold values (ω).

Threshold/Representation		*ω* = 0	*ω* = 0.1	*ω* = 0.2	*ω* = 0.3	*ω* = 0.4	*ω* = 0.5	*ω* = 0.6	*ω* = 0.7	*ω* = 0.8	*ω* = 0.9
Simple	sens.	0,8343	0,6346	0,4694	0,376	0,2972	0,2325	0,1986	0,1692	0,1287	0,1023
	fpr	0,2995	0,0694	0,0304	0,017	0,0092	0,005	0,0035	0,0025	0,0014	0,001
Weighted Content	sens.	0,8393	0,6565	0,5203	0,4423	0,38	0,3163	0,2707	0,215	0,1769	0,1198
	fpr	0,1972	0,0482	0,0225	0,0134	0,0088	0,0058	0,0043	0,0028	0,002	0,0012
Weighted Structure	sens.	0,8511	0,7112	0,5944	0,5015	0,4429	0,3617	0,3145	0,2523	0,2033	0,1492
	fpr	0,1864	0,0523	0,0253	0,0159	0,0085	0,0055	0,004	0,0026	0,0019	0,001
Weighted Hybrid	sens.	0,8605	0,7219	0,6023	0,5093	0,4516	0,3697	0,3209	0,2625	0,2099	0,1549
	fpr	0,1812	0,0502	0,0231	0,0148	0,0086	0,0056	0,0041	0,0027	0,0018	0,001
Protein-Term	sens.	0,9997	0,6763	0,5156	0,4566	0,3894	0,3346	0,3079	0,2402	0,1833	0,1274
	fpr	0,3723	0,0381	0,0166	0,009	0,0069	0,0052	0,0046	0,003	0,0022	0,0011
FFC	sens.	0,9763	0,6073	0,4358	0,3317	0,2972	0,2621	0,2325	0,1672	0,1441	0,1185
	fpr	0,4431	0,0553	0,0214	0,0091	0,0057	0,0046	0,0038	0,002	0,0018	0,0011

We can see from the results shown on Figures 4–8 and Tables 5–9 what we previously stated about the influence of the entropy value. As expected the more complex representations (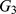 or ProteinTerm and 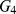 or FFC graph) have higher entropy values which implicitly increases the Sensitivity and fpr values (by increasing the FP and decreasing FN). The opposite holds for the simpler representations (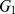 or Simple and 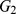 or Weighted graph).

If we average the AUC values for a single algorithm over all graph representations (Table 10) the top ranking algorithm is the edge clustering with 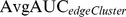 = 0.9065, followed by 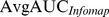 = 0.8963, 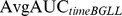 = 0.8913, 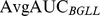 = 0.8864, and 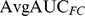 = 0.8831. This result is in line with the well known fact that protein interaction networks have many multifunctional proteins that perform several functions, and are expected to interact specifically with distinct sets of partners, simultaneously or not, depending on the function performed. If we look in more detail at Tables 5–9 we can get a better perspective about the quality of the different annotation process based on each of the clustering algorithms.

**Table 10:** Values for the AUC for the functional annotation with each clustering algorithm and graph representations for the PIN and the average AUC values per algorithm and per representation.

Clustering Alg./Representation	FC	BGLL	TimeBGLL	EdgeCluster	Infomap	AvgAUC
Simple	0,8432	0,8446	0,8653	0,8789	0,8589	0,8581
Weighted Content	0,8733	0,8740	0,8757	0,8902	0,8730	0,8771
Weighted Structure	0,8835	0,8868	0,8913	0,9046	0,8886	0,8909
Weighted Hybrid	0,8882	0,8917	0,8975	0,9107	0,8928	0,8961
Protein-Term	0,9267	0,9341	0,9210	0,9420	0,9660	0,9379
FFC	0,8839	0,8875	0,8979	0,9129	0,8986	0,8962
AvgAUC	0,8831	0,8864	0,8913	0,9065	0,8963	

Table 11 shows the corresponding sensitivity and false positive rate values for each of the algorithms combined with each of the representations at a fixed threshold *ω* = 0. These values are a general indicator of the behaviour of the corresponding annotation process. The EdgeCluster algorithm shows much greater false positive rate as compared to the next in line (according to AvgAUC) Infomap. In fact, Infomap has the overall lowest levels of false positive rates with any graph representation. This means that Infomap performs very stringent clustering of the PIN which results in clusters that are poor in terms of function (term) diversity therefore missing out on part of the functions (terms) which should be associated with a query protein. This leads to a very precise, but incomplete view of the annotation set of the query protein. On the other hand EdgeCluster, timeBGLL, BGLL, and FC achieve much higher sensitivity at the price of a high false positive rate, which means that the annotation set view is much richer but more noisy as compared to Infomap. All of these results are due to the fact that the ratio between the number of clusters generated with Infomap and the other algorithms (all have similar numbers of clusters) is approximately 2.5∶1.

**Table 11:** Values for the sensitivity (sens.) and the false positive rate (fpr), for the functional annotation for each graph representation using each of the clustering algorithms, at a fixed threshold value (*ω* = 0).

Clustering Alg./Representation		FC	BGLL	TimeBGLL	EdgeCluster	Infomap
Simple	sens.	0,8343	0,7166	0,8185	0,8184	0,7523
	fpr	0,2995	0,0447	0,1724	0,1403	0,0614
Weighted Content	sens.	0,8393	0,8381	0,8220	0,8992	0,7757
	fpr	0,1972	0,1942	0,1525	0,2740	0,0547
Weighted Structure	sens.	0,8511	0,8524	0,8426	0,9171	0,8034
	fpr	0,1864	0,1833	0,1376	0,2613	0,0536
Weighted Hybrid	sens.	0,8605	0,8615	0,8539	0,9232	0,8104
	fpr	0,1812	0,1786	0,1389	0,2537	0,0531
Protein-Term	sens.	0,9997	0,9992	0,9995	0,9997	0,9999
	fpr	0,3723	0,3416	0,3927	0,4104	0,1456
FFC	sens.	0,9763	0,9693	0,9573	0,9823	0,9636
	fpr	0,4431	0,4361	0,3492	0,5042	0,4164

The performance of the algorithms on the different graph representations proposed in this research is consistent in all the experiments as can be seen in Table 10. As expected the simple graph representation (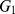) has the lowest AUC values for all clustering approaches. The hybrid weighting scheme (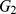) outperforms each of the separate content and structure weighting, with structure being more informative than the content. The rise in performance noted when using the FFC graph representation (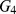) suggests that the actual PIN is lacking part of the real interactions that occur between pairs of proteins. Finally, the Protein-Term representation (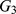) yields the best results in terms of AUC, but both 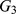 and 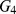 have the noisy annotation problem as stated before (even for the usually low noise Infomap algorithm). In terms of complexity it is clear from Tables 1 and 2 that the 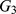 and 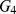 representations are more complex and this computational complexity should be taken into account when deciding on the appropriate representation for a PIN. Also a network wide annotation would be very impractical if we use 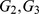, or 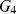, since the clustering algorithm needs to be run for every query protein. On the other hand a scenario in which a wider set of possible annotations needs to be determined for a single (or a few) protein(s) would greatly benefit from these augmented PIN graph representations.

In summary and considering the goals defined our results show that all of the proposed novel representations yield a significant improvement in the function prediction performance over the simple unweighted graph representation. The Protein-Term graph representation is the most informative one and if computational resources are not scarce it is the representation that should be used for the prediction. The next in line is the FFC graph representation, followed by the hybrid weighted graph representation. The ease of further augmentation of these two representation (for example with similarity metrics based on GO instead of using a simple Jaccard index) is their added value and they can be further improved to maximize the annotation prediction performance. All of the clustering algorithms used in this paper perform very good on the PIN, as it was shown in the clustering validation section, with Infomap being the best in that context. In terms of using these clustering algorithms in the function prediction the most accurate one is the Infomap algorithm, while edgeCluster and timeBGLL have the highest coverage.

As a final note we point out to another potential problem in the process of function prediction using clustering, namely the completeness. It has been estimated that the complete *S. cerevisiae* network has between 37800 and 75500 protein interactions [59]. Currently there are between 55000 and 60000 interactions contained in publicly available repositories for *S. cerevisiae*, which means there are potentially unknown regions of the network which can explain the high false positive rates and low sensitivity stated before.

## Conclusions

Complex protein interaction networks reveal graph properties that can be analysed in terms of functional modules associated with the biological function they perform. In our work we investigated the power of the novel algorithm for complex network clustering combined with novel graph representations of the protein interaction networks, and assess their possibilities for protein function prediction via clustering. We show that using these algorithms we can gain significant knowledge for the modular structure of the network. As these networks carry not only interaction information but also annotations the different representations we propose augment to the prediction process by including this information in the clustering of the network.

The results from our experiments validate the augmented graph representation approach. Even the simplest augmentation i.e. the different weighted graph representations of the PIN significantly improve the results of the function prediction. Our experiments were performed using the simple normalized Jaccard Index as a weighting factor and we are confident that results can be even further improved using a more sophisticated weighting scheme. We used the same weighting when we further augmented the graph representation by adding artificial edges to take into account the well known fact that protein interaction networks to this date are still not completely captured by the experimental methods used for their construction. This representation is very complex and is computationally exhaustive but the potential of uncovering new knowledge is significantly increased. Our experiments showed that the most informative representation is the one where we generate a graph in which every single term associated with a protein becomes a node and the association of proteins and terms is represented by adding an edge between each pair. The power of unravelling the functions of a query protein of this representation is the greatest of all proposed representations, but also the same holds for the computational complexity.

In general if one would like to perform a network wide annotation, usage of the weighted graph representations would be recommended, while the exploration of a single protein, or a small group of proteins, should the performed using either the full functional connected graphs or the protein-terms graph. In terms of selecting a clustering algorithm our results showed that Infomap has the best performance in determining the modular structure of a PIN and is also the most accurate of all tested algorithms. However, the high accuracy comes with the price of low coverage (i.e. the inability to discover a larger set of functions associated with a query protein). The opposite holds for the timeBGLL and EdgeCluster algorithms. Depending on the required results one can choose either a random walk and map algorithm (Infomap) if the priority is to get a narrow set of accurate protein functions, or either an edge clustering/overlapping clusters algorithm (EdgeCluster) or a multi-resolution algorithm (timeBGLL) if coverage of the possible functions is of bigger importance.

## Supporting Information

File S1 **Matlab code for generation of the graph representations.**
(ZIP)
